# The PRRSV-Specific Memory B Cell Response Is Long-Lived in Blood and Is Boosted During Live Virus Re-exposure

**DOI:** 10.3389/fimmu.2020.00247

**Published:** 2020-02-18

**Authors:** Michael C. Rahe, Cheryl M. T. Dvorak, Abby Patterson, Michael Roof, Michael P. Murtaugh

**Affiliations:** ^1^Department of Veterinary and Biomedical Sciences, University of Minnesota, St. Paul, MN, United States; ^2^Boehringer Ingelheim Animal Health USA, Inc., Ames, IA, United States

**Keywords:** immune memory, PRRSV, B cells, neutralizing antibodies, anamnestic, immunity

## Abstract

Porcine reproductive and respiratory syndrome virus (PRRSV) is an important pathogen of swine health and well-being worldwide largely due to an insufficient understanding of the adaptive immune response to infection leading to ineffective PRRSV control. The memory and anamnestic response to infection are critical gaps in knowledge in PRRSV immunity. The lack of effective tools for the evaluation of the memory response previously hindered the ability to effectively characterize the porcine memory response to infection. However, the creation and validation of a PRRSV nsp7-specific B cell tetramer now facilitates the ability to detect very rare memory B cells and thus define the memory response of the pig. Here, we describe the PRRSV nsp7-specific B cell response following vaccination and challenge in six key secondary lymphoid organs including the identification of PBMCs as the tissue of interest for the memory immune response in pigs. Following live virus challenge of immune animals, an anamnestic response of nsp7-specific memory B cells and neutralizing antibodies was observed. This characterization of the functional humoral immune response to PRRSV answers key questions involved in regional specialization of the immune response following intramuscular inoculation of PRRSV MLV.

## Introduction

Porcine reproductive and respiratory syndrome virus (PRRSV) is one of the most important adversaries of swine health and well-being worldwide. Observed more than 30 years ago, there is still no effective intervention for inducing the development of a broadly protective immune response against the virus. While the reasons for this failure are multivariate, a large part of this deficiency lies in an incomplete understanding of fundamental aspects of the humoral immune response to PRRSV. This component of the adaptive immune response is made up of circulating antibodies and memory B cells. While antibodies are the effector mechanisms, the induction of a memory response is necessary for the development of immune protection, as upon antigen recognition, memory B cells rapidly boost antibody titers with high affinity, isotype switched antibodies.

There has been considerable research focused on the identification and characterization of the antibody response to PRRSV. This work has shown that infection can induce neutralizing antibodies against homologous virus but only partial protection against heterologous challenge (1–7). Unfortunately, the impressive mutability of PRRSV continues to lead to remarkable strain variation resulting in the frequently observed clinical problem where animals which are previously vaccinated, or exposed and recovered, are susceptible to reinfection with new mutated strains of the virus (8–10). Previously exposed animals will clear the virus more rapidly and will not show clinical signs as severe as naïve animals; however, the mechanism by which this partial protection is achieved is unclear (11–13). The identification of broadly neutralizing antibodies, which can markedly reduce viremia following heterologous challenge, would suggest that antibodies play a crucial role in partial protection (14–16). Research characterizing the development and structure of these antibodies, as well as the antigens and epitopes that they recognize, has been insufficient.

Recent work in HIV immunity has shown that memory B cells hold the key to identifying both neutralizing antibodies as well as the epitopes which they bind (17–19). Remarkably, the memory B cell and anamnestic response to PRRSV challenge has largely gone unstudied. Initial work attempted to characterize anatomic regional variation using ELISPOTs to multiple antigens (N, GP5 3′, and nsp2) (20). This study was strong in its design and scope of PRRSV antigens but it was limited by a dearth of reagents and tools to differentiate between antibody secreting cells and true memory cells. The creation of a PRRSV nsp7-specific B cell tetramer has made it possible to eliminate antibody secreting cells from analysis, and thus an in depth investigation of the antigen-specific B cell response to viral infection is now possible (21).

The nsp7 B cell tetramer consists of four biotinylated nsp7 proteins linked together via a streptavidin core which is coupled to phycoerythrin (PE), a bright fluorophore. Upon incubation with suspended B cells, this presents four opportunities for the surface immunoglobulin of B cells to bind the reagent (21). Once bound, there are three other antigens which may be bound by additional surface immunoglobulins of the B cell, resulting in a highly avid reagent. However, because nsp7-specific B cells within tissue are very rare, ~1 in 100,000 B cells, a magnetic bead enrichment step is necessary to concentrate and enumerate tetramer bound cells (22).

Here, we used the nsp7 B cell tetramer to probe six key immune tissues to characterize the development and subsequent involution of the PRRSV-specific B cell response after modified live virus (MLV) vaccination. Following clearance of the virus, we identified the circulating pool of nsp7-specific memory cells and stimulated an anamnestic response in this cell population with a wild type challenge virus (WT) and revaccination with modified live virus. Characterization of the anamnestic neutralizing antibody response to three genetically distinct PRRS viruses showed that previously vaccinated animals that were challenged with live virus developed neutralizing antibodies quicker than PRRSV naïve animals that were challenged with the same virus. This suggests that memory B cells play an important role in heterologous protection. Collectively, the presented data identify circulating PRRSV-specific memory B cells and show the importance of the porcine memory B cell in conferring quick and adaptable humoral immunity against PRRSV re-infection.

## Materials and Methods

### Animal Studies and Tissue Collection

The animal study was funded by Boehringer Ingelheim Animal Health USA, Inc. (BIAH USA), approved by Veterinary Resources' Inc. (Ames, IA) animal care and use committee and carried out under their purview. Conventional cross-bred commercial pigs of ~3 weeks of age that were ELISA and PCR negative for PRRSV and ELISA negative for *Mycoplasma hyopneumoniae* were enrolled in the study and housed in BIVI animal facilities.

The first part of the study was to examine the changes in nsp7-specific B cell kinetics over time in different lymphoid tissues following vaccination. On day 0, 18 animals were inoculated intramuscularly (IM) in the right side of the neck with 2 ml of Ingelvac PRRSV MLV according to manufacturer instructions and were designated as the vaccinated group of animals. Another six animals were designated as PRRSV naïve and were administered 2 mls of PBS (phosphate buffered saline) IM in the right side of the neck and were housed in a separate group from vaccinated animals. On days 0, 7, 14, 28, 56, and 113 three animals from the vaccinated group and one animal from the naïve group were euthanized. Prior to euthanasia, animals were sedated, 80 mls of whole blood was collected in EDTA collection tubes for PBMC isolation and 20 mls of blood was collected in serum separator tubes for serological testing. Immediately post-euthanasia, the following tissues were collected: spleen, one superficial inguinal lymph node (SiLN), one tracheobronchial lymph node (TBLN), one mesenteric lymph node (MLN), and the palatine tonsils. Spleens were sectioned into 3 cm wide strips and all tissues were placed in complete RPMI tissue culture media (RPMI 1640 with L-glutamine, 5% fetal bovine serum (FBS), 10 mM HEPES pH 7.2, 1% MEM non-essential amino acid solution, 1 mM sodium pyruvate, and 20 μg/ml gentamycin) and shipped on ice overnight to the Murtaugh lab at the University of Minnesota, St. Paul, MN.

The second part of the study examined the anamnestic response of vaccination to challenge in various lymphoid tissues. On day 0, 43 animals (vaccinated group) were inoculated IM in the right side of the neck with 2 ml of Ingelvac PRRSV MLV (serial number #RD-PRRSV01). Twenty-eight animals (PRRSV naïve group) were administered 2 mls of PBS IM in the right side of the neck and were housed in a separate group from vaccinated animals. At day 113, animals were further inoculated according to Table 1. Control animals, three vaccinated and three naïve animals (Groups 1 and 5), were not inoculated on day 113. For the PRRSV 1-7-4 challenge group, six vaccinated and six naïve animals (Groups 2 and 6, Table 1) were challenged with an infectious PRRSV field strain with an RFLP type of 1-7-4 (provided by BIAH USA) intranasally (1 ml per nostril) and injected with 1 ml IM in the left side of the neck for a total dose of 5.54 log TCID_50_/dose. For the nsp7 inoculated group, five vaccinated and five naïve animals (Groups 3 and 7) were administered 100 μg of nsp7 recombinant protein mixed 1:1 with AddaVax, a squalene-based oil-in-water nano-emulsion (InvivoGen). For the MLV group, six vaccinated and six naïve animals (Groups 4 and 8) were inoculated with 2 ml of Ingelvac PRRS MLV (Boehringer Ingelheim Animal Health USA, Inc.) in the left side of the neck. A total of 20 pigs, 1–3 animals per group, were euthanized on day 118 and another 20 on day 133 as indicated in Table 1. Blood and tissues were collected and shipped as described above.

**Table 1:** Treatment groups for the anamnestic experiment.

**Group**	**N**	**Vaccinated on day 0**	**Treatment on day 113**	**# of animals sacrificed on day 118**	**# of animals sacrificed on day 133**
1	3	Yes	None	2	1
2	6	Yes	PRRSV 1-7-4	3	3
3	5	Yes	nsp7 protein	2	3
4	6	Yes	MLV	3	3
5	3	No	None	1	2
6	6	No	PRRSV 1-7-4	3	3
7	5	No	nsp7 protein	3	2
8	6	No	MLV	3	3

Upon receipt, serum was separated and stored at −20°C, whole blood was immediately used for PBMC isolation, and tissues were immediately processed for single cells. Whole blood was diluted 1:2 with 1× PBS. Diluted blood was then slowly layered over lymphocyte separation media (LSM, Mediatech, Inc., Manassas, VA) at a 1:3 ratio in a 50 ml conical tube. Samples were centrifuged at 2,000 RPM (900 × g) at 20°C for 30 min with the brake off. Following centrifugation, the PBMC layer was aspirated, transferred to a new tube, and washed in 1× PBS. Following centrifugation at 1,400 RPM (450 × g) for 10 min, pelleted cells were resuspended in ACK lysis buffer (Lonza, Walkersville, MD) at a dilution of 1:6 pellet to ACK to lyse remaining red blood cells. Samples were incubated, with gentle rocking, for 10 min. ACK was then diluted 1:2 with complete RPMI and centrifuged at 1,400 RPM (450 × g) for 10 min. Pelleted cells were resuspended in complete RPMI and counted with a hemocytometer. Samples were centrifuged at 1,400 RPM (450 × g) for 10 min and then resuspended in freezing media (50% FBS, 40% complete RPMI, 10% DMSO), aliquoted into 2 ml freezing tubes (20–40 million cells/tube), placed in −80°C overnight and then transferred to liquid nitrogen storage the next day.

Solid tissue was processed as previously described with the following modifications (22). Lymph nodes and tonsil were scored with a sterile razor blade prior to cellular dissociation using the plunger of a syringe. Following ACK lysis resuspension and incubation, ACK lysis buffer was diluted 1:2 with complete RPMI + 5% FBS media. Cells were frozen in liquid nitrogen between 20 and 40 million cells/tube depending upon the cellular yield from each tissue.

### PRRSV Viremia and Antibody Levels

Detection of viral RNA in serum samples was performed by Boehringer Ingelheim Animal Health USA, Inc. (St. Joseph, MO) using a PRRSV RT-qPCR assay with a cut-off of 9 copies/ml.

Serum antibody testing was performed by Boehringer Ingelheim Animal Health USA, Inc. (St. Joseph, MO) for examination of PRRSV nucleocapsid antibodies using the PRRSV X3 ELISA (IDEXX Laboratories, Inc., Westbrook, ME) (23). Data is shown as an S:P ratio with a cut-off for antibody presence >0.4.

An nsp7 IgG ELISA was carried out as previously described (21). Serum was diluted 1:50 in PBS and the cutoff for positive samples was established at an OD >0.1. The nsp7 avidity ELISA was performed as previously described, using nsp7 as the antigen (24). Only nsp7 ELISA positive samples were analyzed using the avidity assay to examine the strength of their nsp7 antigen-antibody interaction. Results are shown as the avidity index (OD_Gn+_/OD_Gn−_).

### Nsp7-Specific B Cell Analysis

The number of nsp7 specific B cells in each of the tissues was determined using an nsp7 tetramer pulldown which was performed, gated, and analyzed as previously described (21).

### PRRSV Serum Neutralization Assay

Serum samples from day 118 and day 133 were analyzed using a PRRSV neutralizing antibody (NA) assay against three genetically diverse PRRS viruses to measure the ability of serum antibodies to inhibit PRRSV infection *in vitro*, as previously described (14). The three PRRSV isolates used were: VR2332 (RespPRRS MLV parental strain, PRRSV2/USA/Lab7, GenBank ID AF066183.4), a historical isolate MN184 (MN184A, GenBank ID DQ176019.1), and a contemporary isolate RFLP 1-7-4 (PRRSV2/USA/Lab3, GenBank ID MN175677). A comparison of these whole genome sequences with that of the MLV vaccine virus (GenBank ID AF159149.1) was performed using BLASTn to determine the percent identity between the isolates.

### Statistical Analysis

Significance was determined by unpaired *t*-test analysis using GraphPad prism software version 8.3.1 (GraphPad Software, LLC). A *p* < 0.05 was considered significant.

## Results

### Kinetics of PRRSV Viremia and Antibody Levels Following Vaccination

**Figure 1:**
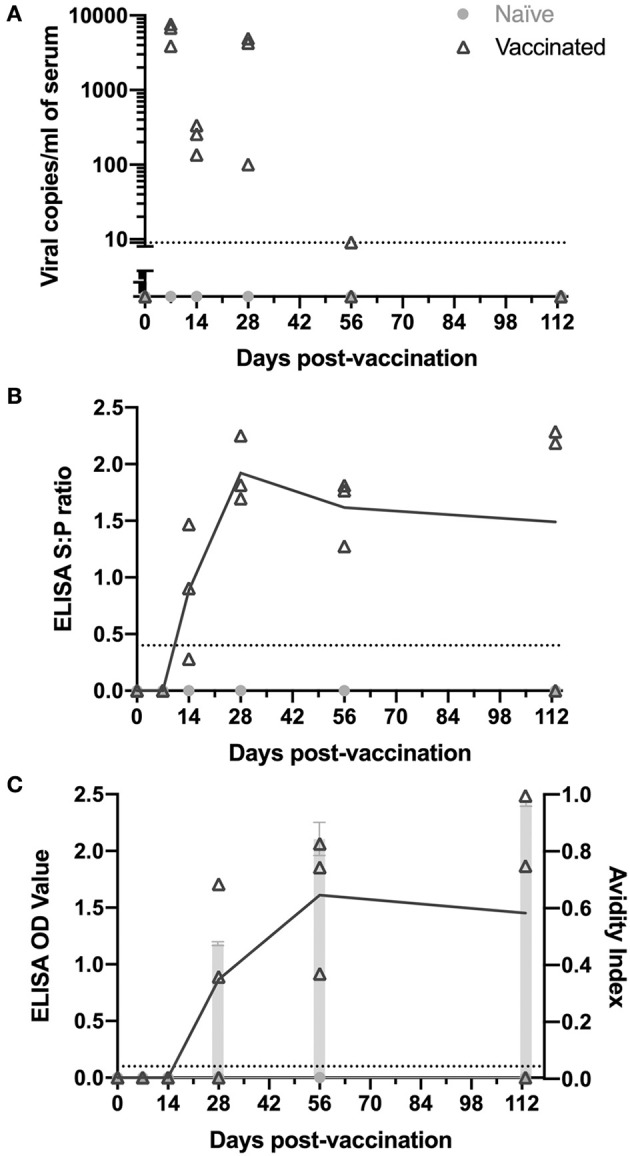
Kinetics of PRRSV viremia and antibodies following vaccination. Animals were vaccinated or not vaccinated at day 0. At each time point (7, 14, 28, 56, and 113 days post-vaccination), three vaccinated and one non-vaccinated animal were sacrificed and serum was obtained. Serum samples were examined for **(A)** PRRSV viremia levels, **(B)** PRRSV antibodies by IDEXX ELISA, and **(C)** nsp7-specific antibodies by ELISA (left y-axis) and avidity index (right y-axis) as shown by light gray bars. Viral copy number and ELISA cut-off values are shown as a dotted line. All naïve animals were non-viremic and antibody negative.

Vaccinated and naïve animals (day 0 treatment) were examined at six time points over 113 days to identify and track the kinetics of viremia and antibody levels. At each time point, three vaccinated and one naïve animal were sacrificed and serum was examined for viremia, PRRSV nucleocapsid antibodies, PRRSV nsp7-specific antibodies, and the avidity of the PRRSV nsp7 antibodies (Figure 1).

Following vaccination, viremia peaked at 7 days post-vaccination, was at the edge of detection by day 56, and was absent at day 113 (Figure 1A). Each time point was examined using different individual animals as the tissues tested at each time point for memory cells required euthanasia of the animals. Thus, the low viremia levels at day 14 and high levels again at day 28 in some animals was not concerning, since it remained present in all animals (Figure 1A). Naïve animals were non-viremic at all time points (Figure 1A).

The presence of PRRSV antibodies were examined using both an IDEXX ELISA, detecting PRRSV nucleocapsid (HerdChek PRRS ELISA package insert; IDEXX Laboratories, Westbrook, Maine) (23), and an nsp7-specific ELISA. Vaccinated animals had detectable circulating anti-PRRSV IgG antibodies as determined by IDEXX ELISA by day 14 post-vaccination (Figure 1B). Antibodies peaked at day 28 and were observed at high levels through day 113 in animals sacrificed at each time point, except one animal at day 113 (Figure 1B). The nsp7 protein is known to be immunogenic, is involved in the humoral immune response and is a good candidate for diagnostics due to its high conservation among PRRSV-2 isolates (25, 26). Antibodies against PRRSV nsp7 were first detected at day 28, 2 weeks later than nucleocapsid antibodies, peaked at day 56, and were observed at high levels at day 113 in 2 of the 3 animals examined (Figure 1C). Interestingly, one of the day 28 vaccinated animals was negative for nsp7 antibodies, but serologically positive against nucleocapsid (Figures 1B,C). At day 113 the nucleocapsid antibody negative pig was also negative for nsp7 antibodies, suggesting this animal either did not receive vaccine or did not mount a lasting immune response (Figures 1B,C). Naïve animals were antibody negative at all time points (Figures 1B,C).

The strength of the nsp7-antibody interaction was measured using an avidity ELISA. The avidity index increased over time with average AI's of 0.48 at day 28, 0.84 at day 56, and 0.96 at day 113 (Figure 1C). This increase in nsp7-antibody affinity over time suggests continuous nsp7 germinal center activity starting between days 14 and 28 and formation of antibodies with extremely high affinity at later time points.

### Kinetics of the nsp7-Specific B Cell Response in Immune Tissues

**Figure 2:**
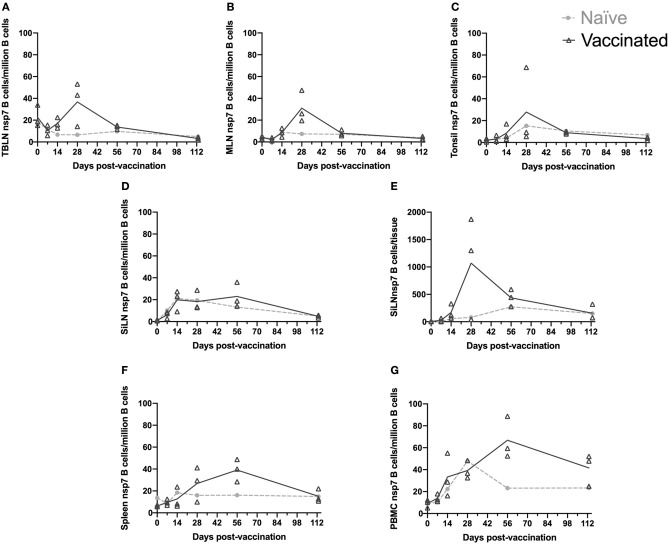
Kinetics of nsp-7 specific B cells in immune tissues. Blood and tissues were harvested from animals that were vaccinated or naïve at day 0 and three vaccinated and one naïve animal were sacrificed at each time point. Nsp-7-specific B-cells were identified using an nsp-7 tetramer pull-down. The number of nsp-7-specific B-cells per million B cells was determined at each time point for **(A)** TBLN, **(B)** MLN, **(C)** tonsil, **(D)** SiLN, **(F)** spleen, and **(G)** PBMCs. **(E)** The number of nsp-7-specific B-cells per tissue was determined at each time point for SiLN.

Characterization of the kinetics of the nsp7-specific B cell population was examined using the nsp7-tetramer to identify nsp7-specific B cells over a time course in various immune tissues (Figure 2). The normalization of collected data across different tissue types and lymph nodes was achieved by dividing the number of live (fixable viability dye^−^), CD21^+^, nsp7 tetramer^+^, and decoy tetramer^−^ cells by the total number of live CD21^+^ B cells that were incubated with the nsp7 tetramer prior to pulldown enrichment and is presented as nsp7-specific B cells/million B cells. The frequency of nsp7-specific memory B cells has been shown previously to be very low and this was confirmed in the tissues examined here (22). Single negative control animals were used for each time point and tissue which were used to compare with vaccinated animals' memory cell response. These PRRSV naïve animals were expected to have nsp7 tetramer specific B cells since recognition of antigen by naïve B cells is necessary for the generation of an antibody response, whereas a true increase in nsp7-specific B cells due to vaccination should be higher than levels observed for this control pig (Figure 2).

The kinetic response of nsp7-specific B cells to PRRSV MLV vaccination within the TBLN, MLN, and tonsil was strikingly similar in timing and comparable in magnitude (Figures 2A–C). The largest increase in nsp7-specific B cell frequencies were observed on day 28 with either one or both of the nsp7 serology positive animals, depending on the tissue. There was high animal to animal variation at day 28 with one of the animals showing high nsp7-specific B cell frequencies in all three tissues, another that was high in TBLN, but low in tonsil, and not surprisingly, the nsp7 seronegative animal was similar to the nsp7-specific B cell frequencies of naïve animals in all three tissues. However, subsequent contraction of this population of nsp7-specific B cells occurred by day 56 with no difference in numbers between vaccinated and naïve animals.

Interestingly, nsp7-specific B cell populations in the SiLN showed no difference between vaccinated and naïve animals (Figure 2D). However, when processing the SiLNs from vaccinated pigs at day 28, the two nsp7 antibody positive animals had markedly enlarged lymph nodes in comparison to the naïve animal or the seronegative vaccinated animal. Using our standard analysis (nsp7-specific B cells/million B cells) no difference in frequency of nsp7-specific B cells between enlarged SiLN and normal SiLN was observed (Figure 2D). However, since nearly all of the cells from the SiLN were able to be examined by flow cytometry due to the small size of the lymph nodes (as were all TBLN and tonsil cells) we were able to examine the number of nsp7-specific B cells in the entire tissue to take into account the enlarged lymph nodes in antibody positive animals. Evaluation of the total number of live nsp7-specific B cells isolated from the SiLN (nsp7-specific B cells/tissue) as opposed to the normalized numbers (nsp7-specific B cells/million B cells) identified a large difference between vaccinated, antibody positive animals with enlarged SiLNs and animals with normal-sized SiLN at day 28 post-vaccination (Figure 2E). Contrary to the results from the SiLN, the TBLN and tonsil showed a similar expansion of nsp7-specific B cells when analyzed either by per million B cells or per tissue (data not shown). The reason for this difference is unclear but may be due to the marked expansion of B cell populations within the SiLN to antigens besides nsp7. As it would not be possible to evaluate all mesenteric lymph nodes or the entire pig spleen, data is presented as nsp7-specific B cells/million B cells for all samples to allow for direct comparison between tissues, corrected for lymphocytosis, and to prevent potential confounding effects of PRRSV on lymphocyte populations (27).

While nsp7-specific B cell frequencies declined in secondary lymphoid organs between day 28 and day 56, frequencies increased in the spleen and PBMCs during this time (day 28–56). In spleen, day 113 B cell frequencies had contracted back to the levels observed in naïve animals, similar to the rest of the lymphoid tissues, but in PBMCs, B cell levels were still elevated similar to day 28 levels (Figures 2F,G). The retention of this population of nsp7-specific B cells within PBMCs 113 days post-vaccination, when all other evaluated lymphoid organs were back to naïve levels, would suggest that these are long lived memory cells.

### Viremia and Antibody Levels Following Challenge

**Figure 3:**
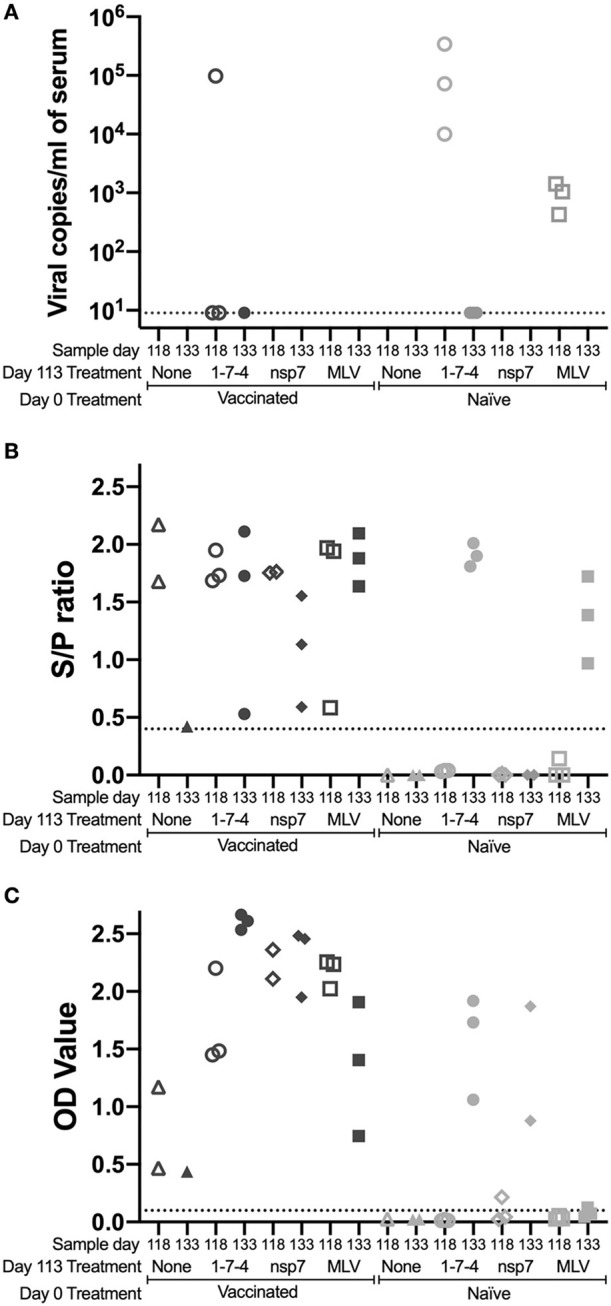
Kinetics of PRRSV viremia and antibodies following day 113 challenge. Animals were vaccinated or not vaccinated at day 0 and given a secondary challenge at day 113. At day 118 and 133, 1–3 animals were sacrificed per group and serum was obtained. Serum samples were examined for **(A)** PRRSV viremia levels, **(B)** PRRSV antibodies by IDEXX ELISA, and **(C)** nsp7-specific antibodies by ELISA. Cut-off values are shown as a dotted line. Day 0 vaccinated animals are shown as dark symbols and naïve animals as lighter symbols. Day 118 samples are shown as open symbols and day 133 samples are shown as closed symbols. Day 113 treatment symbols are designated as a triangle for no treatment, a circle for 1-7-4 challenge, a diamond for nsp7 inoculation, and a square for MLV vaccination.

The previously vaccinated or naïve animals (day 0) were challenged at day 113 to examine the anamnestic response to PRRSV. Animals were sacrificed at either day 118 (5 days post-challenge) or day 133 (20 days post-challenge). Serum from challenged and control animals was evaluated for viremia at day 118 and 133 (Figure 3A). In previously vaccinated animals, viremia was only observed in 1-7-4 challenged animals and in two of those animals viral levels were barely above the detection threshold of the assay (Figure 3A). In naïve animals, viremia was observed at day 118 (5 days post-inoculation) in all animals that were inoculated with virus (1-7-4 or MLV) and viremia was cleared by day 133 (20 days post-inoculation). This was a similar response to what was observed in animals that were vaccinated with MLV on day 0, although some of those pigs remained viremic at day 28 post-vaccination (Figures 1A, 3A).

PRRSV antibodies were examined following day 113 challenge at day 118 and day 133 using both the IDEXX ELISA and the nsp7-specific ELISA (Figures 3B,C). On the day of challenge (day 113), antibodies were at high levels in vaccinated animals and were absent in naïve animals (Figures 1B,C). Antibodies against both nucleocapsid (IDEXX) and nsp7 were observed in all previously vaccinated animals at day 118 and 133, although high animal to animal variation was observed (Figures 3B, C). Vaccinated animals that were re-inoculated with MLV at day 113 had significantly higher nsp7-specific antibody levels than previously vaccinated animals that had no day 113 treatment (*p* < 0.05) (Figure 3C). This rapid and robust boosting of antibody titers was indicative of an anamnestic response to MLV.

In naïve animals, day 113 virus challenge (1-7-4 or MLV) induced antibodies detectable by IDEXX ELISA by day 133 (Figure 3B). This was similar to the timing of antibodies following day 0 vaccination which were first observed at day 14 post-vaccination (Figure 1B). In the naïve pigs, nsp7-specific antibodies were detected in the 1-7-4 challenge and nsp7 inoculation groups at day 133, but were only observed at low levels in one animal after MLV vaccination (Figure 3C). Retrospective evaluation of the nsp7 response against day 0 MLV vaccination showed that nsp7-specific antibodies were not observed until 28 days post-vaccination, so this low level nsp7 antibody response at day 133, 20 days post-vaccination, was expected (Figures 1C, 3C).

A comparison of day 133 antibody levels between previously vaccinated and naïve animals with the same day 113 treatment showed significantly higher levels in previously vaccinated animals after 1-7-4 challenge (*p* < 0.05) or MLV vaccination (*p* < 0.05) (Figure 3C). Higher antibody levels were also observed in previously vaccinated animals as compared to naïve animals following nsp7 treatment (Figure 3C).

### Memory B Cell Frequency in PBMCs and Tissues

**Figure 4:**
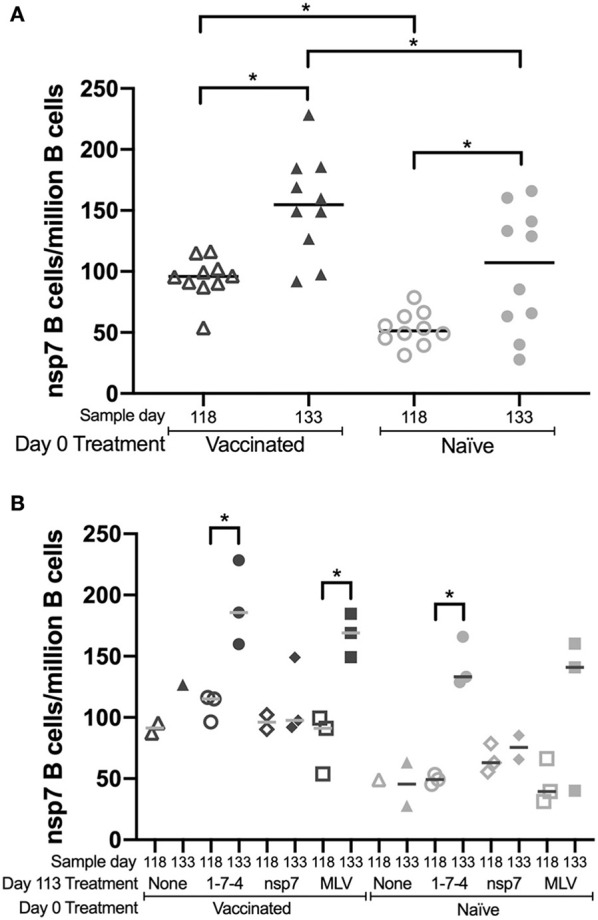
Memory B cell presence in PBMCs following day 113 challenge. Animals were vaccinated or not vaccinated at day 0 and given a secondary challenge at day 113. At day 118 and 133, 1–3 animals were sacrificed per group and PBMCs were obtained. The number of nsp7-specific B cells in PBMC populations was determined using an nsp7 tetramer. At day 118 and 133 the number of nsp7-specific B cells/million B cells was compared between **(A)** all animals from the day 0 vaccinated or naïve groups (4 groups at day 113 with 1–3 animals per group for a total of 10 naïve and 10 vaccinated animals at each time point) and **(B)** each of the different day 113 treatments. Day 0 vaccinated animals are shown as dark symbols and naïve animals as lighter symbols. Day 118 samples are shown as open symbols and day 133 samples are shown as closed symbols. Day 113 treatment symbols are designated as a triangle for no treatment, a circle for 1-7-4 challenge, a diamond for nsp7 inoculation, and a square for MLV vaccination. *Statistically significant differences between groups are shown.

Nsp7 antigen-specific B cell frequencies in PBMCs were evaluated on days 118 (5 dpc) and 133 (21 dpc), as the kinetic study had identified an nsp7 antigen-specific B cell population within PBMCs (Figure 2G). On day 118 and day 133, there were significantly more nsp7-specific B cells in PBMCs from day 0 vaccinated animals than in naïve animals (Figure 4A). A significant increase in the number of nsp7-specific B cells was also observed from day 118 to 133 in both vaccinated and naïve animals (Figure 4A).

Analysis of the SiLN, TBLN, MLN, tonsil, and spleen at day 118 in previously vaccinated animals showed no expansion of nsp7-specific B cell populations (data not shown). PBMCs from animals that were exposed to live virus at day 113 (174 and MLV groups) showed significant expansion of nsp7-specific B cell populations from day 118 to 133 regardless of day 0 treatment, except for day 0 naïve/day 113 MLV treated animals (Figure 4B). One of the naïve animals that was MLV vaccinated at day 133 showed naïve levels of nsp7-specific B cells, but the other two showed expansion of B cells, similar to the other live virus treated animals (Figure 4B).

Interestingly, similar kinetics were observed for nsp7-specific B cells in PBMCs and nsp7 antibody levels over time (Figures 1C, 2G) and for most of the day 113 treatments, similar trends were observed in both day 0 vaccinated and naïve animals (Figures 3C, 4B). A comparison between nsp7-specific antibody levels in serum and nsp7-specific B cells in PBMCs using all animals with a positive ELISA value (OD > 0.1) from the kinetic and anamnestic studies was performed showing no significant correlation between nsp7-specific antibodies and nsp7-specific B cell numbers in PBMCs. Thus, nsp7 IgG antibody levels were not predictive of nsp7-specific B cell numbers in PBMCs.

### PRRSV Neutralizing Antibody Titers

Characterization of the humoral anamnestic response to PRRSV challenge would be incomplete without evaluation of the functional neutralizing antibody response. Because of the impressive strain variability of PRRSV, we evaluated the generation of neutralizing antibodies in our anamnestic study against three genetically diverse PRRS viruses: VR2332 (99.9% identity to MLV, GenBank ID AF159149.1), MN184 (88.0% identity to MLV), and a 1-7-4 field virus (85.8% identity to MLV) (15).

**Figure 5:**
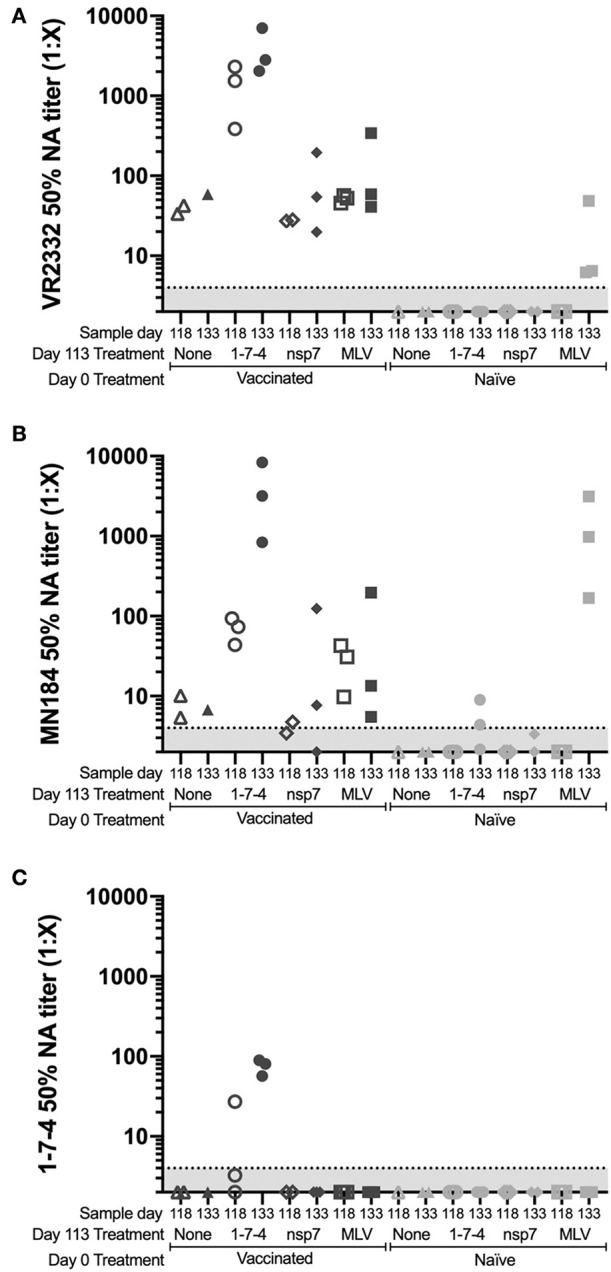
Neutralizing antibody titers against three diverse PRRS viruses following day 113 challenge. Animals were vaccinated or not vaccinated at day 0 and given a secondary challenge at day 113. At day 118 and 133, 1–3 animals were sacrificed per group and serum was obtained. The 50% neutralizing antibody titers against **(A)** VR2332, **(B)** MN184, and **(C)** 1-7-4 are shown. The assay can detect viral titers of 1:4 or greater, below which is shown shaded in gray. Day 0 vaccinated animals are shown as dark symbols and naïve animals as lighter symbols. Day 118 samples are shown as open symbols and day 133 samples are shown as closed symbols. Day 113 treatment symbols are designated as a triangle for no treatment, a circle for 1-7-4 challenge, a diamond for nsp7 inoculation, and a square for MLV vaccination.

The PRRSV VR2332 strain is the precursor to the MLV vaccine, thus it was expected for MLV vaccinated animals to develop neutralizing antibodies (NA) against VR2332. All day 0 vaccinated animals had NA titers against VR2332 and none of the naïve animals were NA positive at day 118 (Figure 5A). No significant difference was observed between day 118 and 133 NA titers against VR2332 (Figure 5A). However, higher NA titers at both day 118 and 133 were observed for animals challenged at day 113 with the 1-7-4 virus than for any of the other animals, suggesting that the 1-7-4 infection resulted in a stronger and more rapid anamnestic response than re-vaccination (Figure 5A). Naïve animals that were vaccinated with MLV at day 113, showed NA titers at day 133, suggesting that neutralizing antibodies can be induced by vaccine by 20 days post-challenge (Figure 5A).

The ability of vaccine to induce cross-neutralizing antibodies was examined by determining the NA titers against two genetically distinct viruses (MN184 and a 1-7-4 contemporary strain). Day 0 vaccinated animals all showed MN184 NA titers at day 118 except one animal which was just below the cut-off of the assay (Figure 5B). MN184 NA titers were generally lower than those against VR2332 (Figures 5A,B). In previously vaccinated animals that were challenged with 1-7-4 virus, MN184 titers increased from day 118 to 133 (Figure 5B). Interestingly, MN184 NA titers were higher than VR2332 NA titers in naïve animals that received MLV on day 113 (Figure 5B). Naïve animals challenged with 1-7-4 virus on day 113 induced MN184 NA titers, whereas VR2332 NA titers were undetectable in these animals (Figure 5B). Not surprisingly, the only animals that induced 1-7-4 NA antibodies were previously vaccinated animals that were challenged with 1-7-4 virus at day 113 (Figure 5C). One animal had detectable 1-7-4 NA titers at day 118 and all three animals were 1-7-4 NA positive at day 133, although all NA levels were lower than NA titers against VR2332 and MN184 (Figure 5C). Only previously vaccinated animals that were challenged with 1-7-4 were able to develop 1-7-4 NA titers showing that vaccination did not induce cross-neutralizing antibodies but may have resulted in a memory response which recognized 1-7-4 neutralizing epitopes thus allowing for the quick increase in NA titers.

## Discussion

The humoral immune response to viral infection is essential for the development of sterilizing immunity. The aim of the presented studies was to characterize the response of both memory B cells and antibodies, the two most important effectors of the humoral immune response, to PRRSV vaccination and challenge. With the use of an nsp7 B cell tetramer, we asked if there was regional variation in the antigen specific immune response to PRRSV vaccination and where immune memory was maintained. We sought to answer these fundamental questions of PRRSV immunology by identifying the kinetics of the nsp7-specific B cell response to PRRSV MLV vaccination in five key secondary lymphoid organs and PBMCs. Nsp7 protein is not known to be important for neutralization of the virus, but does produce a long-lived humoral antibody response that can be used to identify immune animals (26).

For all of the studied lymph nodes, day 28 was the high point for nsp7-specific B cells within the total B cell population. This rapid proliferation of nsp7-specific B cells was indicative of germinal center formation and corresponded with the first observed nsp7-specific IgG in serum. By day 56, the number of nsp7-specific B cells had contracted within the lymph nodes, but the frequency of these cells increased in the spleen as well as within the PBMC B cell populations. The reason for the decline of these antigen specific populations in the lymph node can be attributed to the involution of germinal centers. The large increase in nsp7-specific B cells in the blood is likely due to the migration of nsp7-specific memory B cells out of the lymph nodes and into blood where these cells would be able to more actively search for specific antigen, as has been observed in other species with several pathogens (28–30). The delayed expansion of nsp7-specific B cells in the spleen is less clear. The ability of PRRSV to maintain itself within lymphoid tissues and potentially stimulate leukocytes within that organ must be considered; however, persistent infection of macrophages by PRRSV has not been shown to occur in the spleen as it has in other lymphoid tissues, such as the superficial inguinal lymph node and tonsil (31). Remarkably, the highest frequency of nsp7-specific B cells was noted in the spleen at day 56; whereas all other lymph nodes had contracted down to naïve levels at this point. This zenith of nsp7-specific B cells in the spleen also corresponded with a marked increase in nsp7 antibody avidity from day 28 to 56 where it was close to maximal AI levels.

The observed nsp7-specific B cell response in the mesenteric lymph nodes of vaccinated animals was noteworthy, as this area of the body is thought to primarily respond to antigens from the intestines. This immune induction corresponds to that observed in previous studies which reported that PRRSV infection induces a PRRSV-specific immune response in the lymphoid tissue corresponding with the alimentary tract (20, 31, 32). The identified response in the mesenteric lymph nodes is most likely due to systemic blood flow and the viremic nature of the pathogen.

Previous studies of the porcine memory B cell, as well as that of the mouse, have shown that memory B cells develop and are maintained in several lymphoid structures (20, 33). However, studies from other species would suggest that at least some component of memory is maintained in circulation (30, 34, 35). In this study, there was no evidence of nsp7-specific cells within key secondary lymphoid tissues after day 56, including day 118, 5 days after challenge. Significantly, an nsp7 antigen-specific B cell population was circulating in the blood at day 56 post-vaccination which was maintained at day 113 post-vaccination. This population of cells was then challenged at day 113 with a virulent 1-7-4 virus, a booster vaccination of MLV vaccine, or nsp7 recombinant protein with adjuvant. While this population of cells did not rapidly expand by 5 days post-challenge, it did significantly increase in animals that were inoculated with a live virus, either 1-7-4 or MLV vaccine virus. It is important to note that even though we observed an increase in nsp7-specific antibodies from animals administered recombinant nsp7 protein with adjuvant, an increase in nsp7 antigen-specific B cells was not observed. This suggests that antigen recognition by memory B cells can result in antibody boosting, but to enhance the memory cell population active infection and replication may be necessary.

Evaluation of neutralizing antibodies to three genetically distinct viruses was carried out to determine the magnitude of the neutralizing anamnestic response following challenge with a 1-7-4 live virus or MLV booster vaccination. We observed that previously vaccinated animals challenged with a virulent virus developed higher neutralizing titers against this virus than naïve animals that were exposed to the same virulent virus. The rapidity of this neutralizing antibody response should ultimately lead to earlier clearance of the virus and explains at least part of the heterologous protection which has been previously described in prior PRRSV challenge studies (36–39). The memory B cell is likely the cause of this swift neutralizing antibody response and further investigation following the path laid out by Goldeck et al. may be rewarding in discovering potentially conserved neutralizing epitopes (40).

While this study is important for laying the groundwork of antigen specific B cell kinetics in the pig, it was hindered by the necessity for cross-sectional sacrifice of animals for tissue collection, thus the same animals were not able to be tracked longitudinally. Additionally, there were small numbers at each time point hindering some statistical analyses although definite trends were observed. In spite of this, we were able to characterize the PRRSV-specific B cell kinetics over time in immune tissues, identify PBMCs as the tissue of interest for porcine memory B cell study, and describe the anamnestic antibody and neutralizing antibody response to both live virus infection and recombinant antigen exposure.

## Data Availability Statement

All datasets generated for this study are included in the article/supplementary material.

## Ethics Statement

The animal study was reviewed and approved by Veterinary Resources' Inc.

## Author Contributions

MCR, AP, MR, and MM conceived the study and participated in its design and coordination. AP and MR ran the animal study. MCR performed experiments and interpreted results. MCR wrote the main manuscript. MM helped interpret results. CD helped write the manuscript and interpret results. All authors read and approved the final manuscript.

### Conflict of Interest

AP and MR are employed by Boehringer Ingelheim Animal Health USA, Inc. The authors declare that this study received funding from Boehringer Ingelheim Animal Health USA, Inc. The funder had the following involvement with the study: personnel helped conceive the idea, participated in study design and coordination, ran the animal study, and approved the manuscript.
